# ReGAIN: a bioinformatics platform for assessing probabilistic co-occurrence between resistance genes in bacterial pathogens

**DOI:** 10.1093/bioinformatics/btag505

**Published:** 2026-07-09

**Authors:** Elijah R Bring Horvath, Mathew G Stein, Matthew A Mulvey, Edgar J Hernandez, Jaclyn M Winter

**Affiliations:** Department of Pharmacology and Toxicology, The University of Utah, Salt Lake City, UT 84112, United States; Independent Researcher, Boise, ID 83709, United States; School of Biological Sciences, The University of Utah, Salt Lake City, UT 84112, United States; Henry Erying Center for Cell & Genome Science, The University of Utah, Salt Lake City, UT 84112, United States; Department of Biomedical Informatics, The University of Utah, Salt Lake City, UT 84108, United States; Department of Pharmacology and Toxicology, The University of Utah, Salt Lake City, UT 84112, United States

## Abstract

**Motivation:**

Multidrug-resistant bacterial pathogens continue to rise globally, yet scalable methods are needed to infer how resistance determinants co-occur across pathogen populations and to quantify conditional dependencies underlying co-occurrence and shared genetic context.

**Results:**

We present ReGAIN (Resistance Gene Association and Inference Network), an open-source platform that applies Bayesian network structure learning to infer probabilistic, conditional dependency relationships among antibiotic resistance, heavy metal tolerance, stress response, and virulence determinants in bacteria. In contrast to pairwise co-occurrence analyses, ReGAIN reports conditional probabilities, relative risks, and absolute risk differences with confidence intervals to prioritize candidate relationships for downstream prioritization. Applied across ESKAPEE pathogens, ReGAIN recapitulated established resistance gene relationships and identified additional candidate patterns consistent with co-selection and shared genetic context. Together, these results support scalable, reproducible population-wide analysis of resistance networks for surveillance, comparative genomics and epidemiology.

**Availability:**

ReGAIN analyses are performed using Python v3.11.5 and R v4.4.1 and is available as open-source software through Bioconda at {https://anaconda.org/bioconda/regain-cli}. Source code and documentation can be found at {https://github.com/ERBringHorvath/regain_CLI}. All genomes used in this publication were downloaded from the National Center for Biotechnology Information database. Large supplementary tables and results data from the ESKAPEE pathogen example network analyses can be downloaded from https://figshare.com/articles/dataset/ReGAIN_command_line_software_and_supplemental_figures_/28959431.

## 1 Introduction

Increasing rates of multidrug resistance in clinically important bacterial pathogens pose a monumental threat to global human health (Prestinaci *et al.* 2015, Klein *et al.* 2019, Centers for Disease Control and Prevention US Department of Health and Human Services 2021, Centers for Disease Control and Prevention US Department of Health and Human Services 2022). Among antibiotic resistant bacteria, those classified as multidrug-resistant (MDR) represent the most challenging health threats (Munita and Arias 2016, Sharma *et al.* 2022), as they can encode extensive genetic machinery to evade anti-infective agents (Diekema *et al.* 2019, Forde *et al.* 2019, Ikhimiukor *et al.* 2022, Bring Horvath *et al.* 2025), thereby significantly limiting treatment options. Continued increases in rates of MDR bacteria has been historically attributed to improper antibiotic use, including misuse for non-bacterial infections and overuse in agriculture (Machowska and Lundborg 2018, Manyi-Loh *et al.* 2018, Hutchings *et al.* 2019, Nandi *et al.* 2023). Moreover, the increase in multidrug resistance is significantly driven by the dissemination and acquisition of resistance genes within microbial communities through horizontal gene transfer (Bischoff *et al.* 2005, de Vries *et al.* 2011, Smillie *et al.* 2011, Stecher *et al.* 2012, von Wintersdorff *et al.* 2016, Lambrecht *et al.* 2019, Hussein *et al.* 2021, Bogri *et al.* 2024). Indeed, many multidrug resistance islands are flanked by transposable elements (TEs), further indicating the mobility of not just small operons, but potentially large genomic islands containing extensive resistance gene diversity (Poole *et al.* 2006, Navas *et al.* 2016, Forde *et al.* 2019, Huyan *et al.* 2020, Baker *et al.* 2024). The ability of bacteria to easily exchange antibiotic resistance genes (ARGs) makes multidrug resistance an ever-evolving problem (D’Costa *et al.* 2011, Hutchings *et al.* 2019). Furthermore, exposure to environmental pollutants, such as heavy metals, combined with genetically encoded heavy metal resistance genes, can aid in ARG transfer through co-selection (Akinbowale *et al.* 2007, Li *et al.* 2017, Zhang *et al.* 2016). To better monitor the spread and dissemination of resistance genes, careful curation of genomic datasets, including isolation source, region, and date should be prioritized, such that region-specific and temporal studies can be accurately performed.

Advancements in whole-genome sequencing offer significant opportunities to understand the evolution of multidrug resistance and map common resistance gene co-occurrence patterns in bacterial species. This is especially important in monitoring patterns of co-occurrence with genes conferring resistance to last-line antibiotics, such as the *mcr*-family of colistin resistance genes (Ordooei *et al.* 2015, Liu *et al.* 2016, Hussein *et al.* 2021). Tools like AMRFinderPlus (Feldgarden *et al.* 2021) and ResFinder (Florensa *et al.* 2022) are effective for identifying antibiotic resistance genes, while tools such as PlasmidFinder (Carattoli *et al.* 2014), ISEScan (Xie and Tang 2017), and TnCentral (Ross *et al.* 2021) can be used to detect plasmids and map mobile genetic elements (e.g. integrases, transposases), that often facilitate horizontal gene transfer of resistance determinants. However, these tools are not designed to investigate the complex interplay of resistance genes contributing to MDR phenotypes. Several *in silico* methods designed to expand the analysis of resistome data have been published. However, these methods generally require extensive computational experience (Li *et al.* 2015, Li *et al.* 2017, Shinde 2022) or focus on gene abundance in metagenomic datasets rather than organism-specific occurrence patterns of genes (Li *et al.* 2015, Dhariwal *et al.* 2021). To the best of our knowledge, there are no publicly available bioinformatic platforms designed to quantify co-occurrence of bacterial resistance and/or virulence gene determinants.

In response to the urgent need to not only identify resistance genes but also catalog patterns of resistance gene co-occurrence, we developed the Resistance Gene Association and Inference Network (ReGAIN) genomic pipeline. Built on the foundational capabilities of the National Center for Biotechnology Information’s (NCBI) AMRFinderPlus (Feldgarden *et al.* 2021) for resistance and virulence gene identification, ReGAIN’s core pipeline applies a robust Bayesian network structure learning approach to elucidate probabilistic co-occurrence patterns indicative of multidrug resistance in bacterial pathogens. Importantly, the ReGAIN pipeline is not limited to resistance and stress response genes; it also extends to virulence determinants through a parallel analysis pathway, offering a broader perspective on antimicrobial defense and bacterial pathogenicity. Designed to be flexible and user-friendly, ReGAIN simplifies the bioinformatic workflow into two core modules: data acquisition/dataset creation (Module 1) and Bayesian network analysis (Module 2). Genomes submitted to the data acquisition module are assessed for the presence of resistance and/or virulence genes. From these results, ReGAIN generates a binary presence/absence data matrix, a metadata file, and a combined results file that includes the contig and nucleotide location of each identified gene. These files can then be passed to the Bayesian network analysis module. Module 2 generates an interactive Bayesian network, as well as probabilistic measurements, including mean conditional probability, relative risk, and absolute risk difference, as well as confidence intervals and standard deviation for each metric. Further distinguishing the ReGAIN pipeline are two post-hoc analyses that compute the bidirectional strength of gene co-occurrence, which offer a quantitative method to explore and interpret asymmetry in gene-gene relationships. These metrics enhance our understanding of resistance gene networks, offering new insights into the dynamics of multidrug resistance.

As proof of concept, Bayesian networks were constructed using publicly available genomic data for both Gram-positive and Gram-negative bacterial ESKAPEE pathogens (*Enterococcus faecium*, *Staphylococcus aureus*, *Klebsiella pneumoniae*, *Acinetobacter baumannii*, *Pseudomonas aeruginosa*, *Enterobacter cloacae*, and *Escherichia coli*), which are responsible for the majority of nosocomial infections worldwide and are often resistant to multiple antibiotics (Szczepanowski *et al.* 2004, Prestinaci *et al.* 2015, Munita and Arias 2016, Manyi-Loh *et al.* 2018, Diekema *et al.* 2019, Selim 2022, Shen *et al.* 2023).

## 2 Methods

### 2.1 Selection of genomes and preparation of example datasets

All genomes were downloaded using the NCBI Datasets command-line interface (Sayers *et al.* 2022). Because public genome availability differed significantly among bacterial species, taxon-specific release-date cutoffs were used to generate contemporary yet computationally tractable genome pools for random sampling. For *S. aureus*, *K. pneumoniae*, *A. baumannii*, and *P. aeruginosa*, genomes released after January 1, 2022, were sufficient to generate proof-of-concept genomic populations. For *E. faecium* and *E. cloacae*, which yielded smaller genome cohorts over the same period, the release-date cutoff was extended to January 1, 2020, to generate adequately sized genomic datasets. Because *E. coli* served as the primary proof-of-concept organism in these analyses, a January 1, 2020 cutoff was also used to generate a sufficiently large genome pool for downstream subsampling analyses. *E. coli* genomes were consolidated into a single source directory, from which random subsets of 100, 500, and genomes were selected and copied into new directories, ensuring that each genome had an equal probability of being selected one or more times across population subsets. For the remaining ESKAPE pathogens, random cohorts of 1000 genomes were selected independently using a custom Python script. For *E. cloacae*, all eligible genomes released after January 1, 2020, were included because fewer than 1000 genomes were available after applying the release-date criterion (335 genomes total after genome quality filtering). Release dates were specified using the ‘--released-after <MM/DD/YYYY>’ parameter in the NCBI Datasets command string. Genome quality and completeness were assessed using checkM2 (Chklovski *et al.* 2023), and only genomes displaying ≥98% completeness and <5% contamination were retained for downstream analysis.

For the ReGAIN Curate performance analysis, a 20-gene mixed cohort comprised of resistance genes and transposable elements was selected from the Ec17R multidrug resistance island encoded on *E. coli* strain U15A plasmid pU15A_A (NZ_CP035721.1) (Forde *et al.* 2019, Bring Horvath *et al.* 2025). The selected genes were extracted and annotated using the NCBI BLAST nr_cluster_seq database. The 123 coding sequences encoded by pU15A_A were predicted using Prodigal (Hyatt *et al.* 2010), and the resulting multi-FASTA file was split into individual FASTA files using SeqForge (Bring Horvath and Winter 2025) for use as query inputs to the ReGAIN Curate pipeline as a large-input stress test to assess computational performance.

### 2.2 Data acquisition and dataset creation

ReGAIN’s data acquisition module utilizes NCBI AMRFinderPlus v4.0.23 (Feldgarden *et al.* 2021) annotations as upstream feature calls and converts the resulting annotations into a binary genome-by-feature matrix. Each AMRFinderPlus-reported feature is represented as either present or absent across individual genomes. Features may include acquired resistance genes, resistance-associated point mutations, and virulence genes for supported organisms (Table 1). For all test datasets, resistance and virulence gene determinants were identified using the appropriate ‘--organism’ flag. ReGAIN modules for data acquisition and dataset creation (Module 1), along with ReGAIN Curate, Extract, Combine, collapse-features, matrix-summary, and network-analysis, were developed in Python v3.11.5 using the Pandas v2.2.0 and Biopython v1.83 libraries. ReGAIN Curate additionally utilizes the NCBI BLAST+ software package (Camacho *et al.* 2009).

**Table 1:** Bacterial species supported through the ReGAIN organism-specific data acquisition pipeline.

Gram-negative organisms
*Acinetobacter baumannii*
*Bordetella pertussis*
*Burkholderia cepacia*, *B. pseudomallei, B. mallei*
*Campylobacter* spp.
*Citrobacter freundii*
*Enterobacter cloacae, E. asburiae*
*Escherichia* (also used for *Shigella* spp.)
*Klebsiella oxytoca*, *K. pneumoniae* (*K. pneumoniae* also used for *K. aerogenes*)
*Neisseria gonorrhoeae, N. meningitidis*
*Pseudomonas aeruginosa*
*Salmonella* spp.
*Serratia marcescens*
*Vibrio cholerae*, *V. vulnificus*, *V. parahaemolyticus*

Gram-positive organisms
*Corynebacterium diphtheriae*
*Clostridioides difficile*
*Enterococcus faecalis*, *E. faecium* (*E. faecium* also used for *E. hirae*)
*Staphylococcus aureus*, *S. pseudintermedius*
*Streptococcus agalactiae*, *S. pneumoniae*, *S. pyogenes* (*S. pneumoniae* also used for *S. mitis*)

Table adapted from the AMRFinderPlus (Feldgarden *et al.* 2021) documentation (https://github.com/evolarjun/amr/wiki/Running-AMRFinderPlus#--organism-option). Users may access this list through ReGAIN by running ‘regain AMR --organism-list’.

Runtime for ReGAIN Module 1.0 varies depending on the number of genomes being queried and the capabilities of the computational system. For moderate-to-large datasets (>250 genomes), the average runtime for Module 1.0 is >24 hours using 8 cores on a M1 MacBook Pro. Runtime for Module 1.0 was substantially improved on a high-performance computing system; using 24 dedicated cores, runtime for 1000 genomes was typically completed in under 2 hours.

### 2.3 Bayesian network analysis, visualization of results, and additional analyses

Module 2, responsible for Bayesian network structure learning, was developed in R v4.4.1 and uses the following R packages: bnlearn v4.8.1 (Scutari 2010), gRain v1.3.13 (Højsgaard 2012), gRbase v1.8.9 (Dethlefsen and Højsgaard 2005), visNetwork v2.1.2, graph v1.82.0, scales v1.3.0, and igraph v1.5.0 (Csardi and Nepusz 2006). Parallelization in R was achieved using the following libraries: foreach v1.5.2, doParallel v1.0.17, and parallel v4.3.1. Principal coordinate analyses (PCoA) were generated using vegan v2.6–4 (Oksanen *et al.* 2022) and ellipse v0.5.0. ReGAIN-generated plots were created using RColorBrewer v1.1–3, ggplot2 v3.4.2, and ggraph v2.2.1. Example Bayesian networks were constructed using 500 bootstraps; data was additionally resampled and fitted to networks 500 times to calculate probability means, confidence intervals, and standard deviation. To avoid divide-by-zero errors, a small additive constant (‘ε-stabilization’, 1e-9) was added to both numerator and denominator when computing relative risks, maintaining the appropriate relative risk scale, while avoiding *inf* values. For each genomic dataset, only genes occurring in ≥5% of genomes were included, except for the 1000-genome *E. coli* network analysis, which utilized a minimum inclusion threshold of 25 occurrences (2.5%) in order to avoid excluding rare but potentially biologically relevant resistance determinants. Across all datasets, genes occurring in ≥95% of a given genomic population (i.e. ubiquitously occurring genes) were excluded to reduce noise in the network. PCoA were performed using the Jaccard measure of similarity.

Runtime for Module 2 varies considerably based on the number of genes and overall dataset complexity. For reference, the example dataset provided in the ReGAIN GitHub repository consists of 502 observations (genomes) of 39 features (genes). This dataset takes approximately 25 minutes to run using 500 bootstraps and 100 data resamples (a total of 148 200 queries) on an M1 MacBook Pro with 6 dedicated cores using the ‘bnS’ ReGAIN pipeline. The ‘bnS’ workflow is recommended for all datasets containing <100 genes, when possible. For larger datasets (≥100 genes), the ‘bnL’ workflow must be employed and high-performance computing resources are recommended. Very large and/or complex datasets of ≥100 genes can be run on a desktop computer with multi-core capabilities; however, the analysis will likely take multiple hours or longer to complete. Because exhaustive computation of dependencies is performed, the ‘bnS’ workflow runtime can be longer than the ‘bnL’ runtime for small datasets. However, the ‘bnS’ analysis may provide better overall resolution. To optimize runtime, self-queries (i.e. Gene A = Gene B) are excluded, such that for *N* genes and *R* resamples, the number of pairwise gene queries can be described as N * (*N—*1) * R.

## 3 Results

### 3.1 Overview of ReGAIN Module 1: data acquisition, dataset creation, and dataset analysis

**Figure 1:**
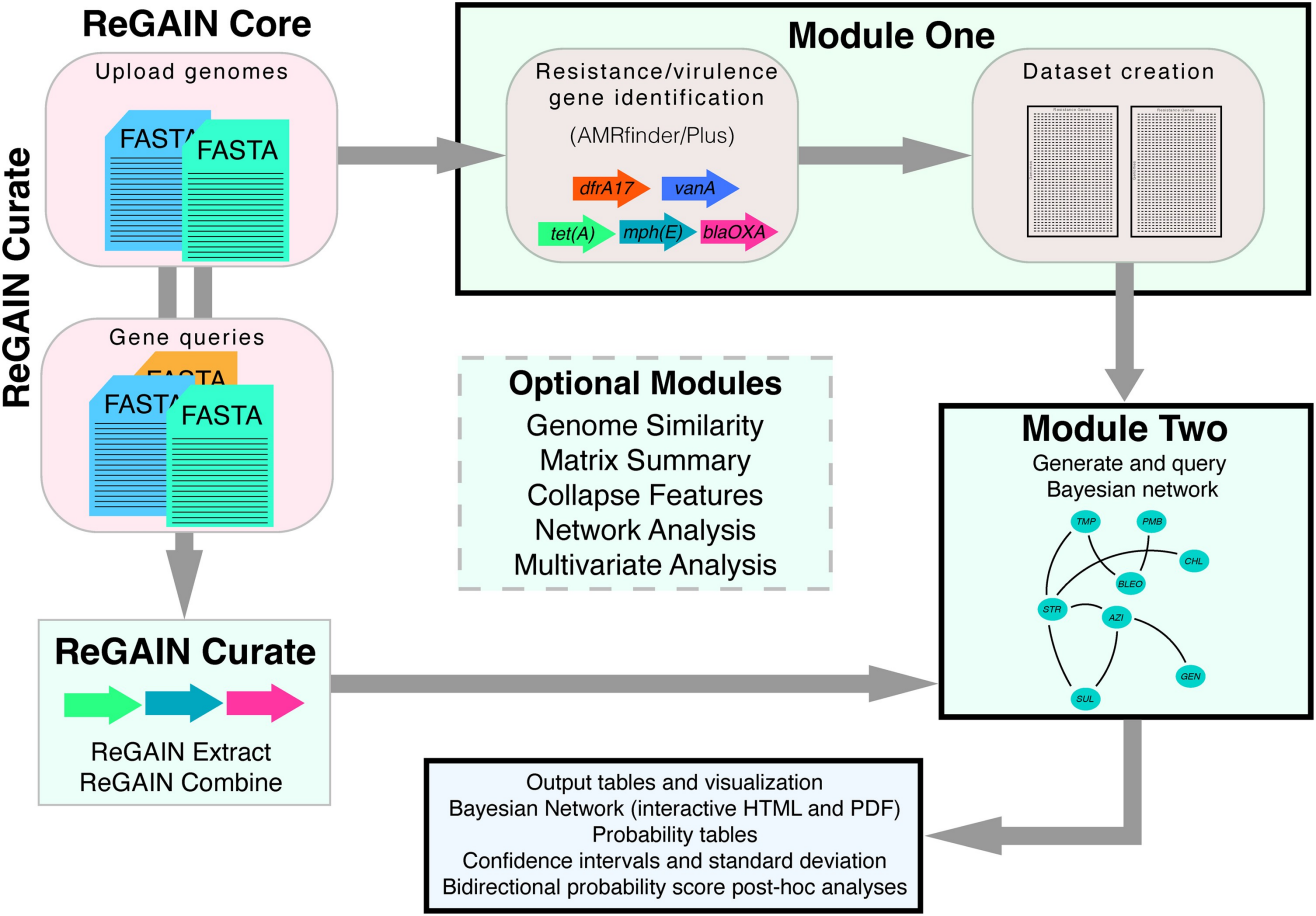
Outline of the ReGAIN bioinformatic pipeline. The first core module uses AMRFinderPlus to identify resistance and/or virulence genes within an input genome population. After all genomes have been processed, Module 1.1, dataset creation, generates two required output CSV files for downstream analysis: a presence/absence matrix of genes across genomes and a metadata file containing all identified genes and their associated gene type, if available. All downstream ReGAIN core analyses can be performed using these two output files. The second core module, Bayesian network structure learning Module 2, utilizes a suggested minimum of 300–500 bootstraps and 100 resamples of the submitted dataset to generate a table of pairwise mean conditional probability, relative risk, and absolute risk difference values for each gene pair in the dataset, including confidence intervals and standard deviation. Additional post-hoc analyses generate bidirectional probability scores based on both conditional probability and relative risk for each gene pair cohort. Finally, an interactive Bayesian network HTML file is generated in parallel with a PDF of the final network. A parallel ReGAIN pipeline, ReGAIN Curate, allows users to query a custom set of genes through a genomic population. Optional modules include multivariate analysis, sequence extraction, dataset combining, workflows to assess average nucleotide (ANI) or Mash distance similarity of an input genomic population, matrix summary statistics, feature binning, and network analysis.

The ReGAIN core pipeline consists of two primary modules: data acquisition/dataset construction and Bayesian network structure learning (Fig. 1). The data acquisition module functions as a wrapper for AMRFinder/AMRFinderPlus and includes both organism-specific and non-specific pipelines. The organism-specific workflow uses AMRFinderPlus to identify known resistance genes, resistance-conferring point mutations, and virulence factors (‘--gene-type resistance’ returns the following AMRFinder-curated bins: AMR, METAL, BIOCIDE, and POINT; ‘--gene-type virulence’ returns the following AMRFinder-curated bins: VIRULENCE, HEAT, ACID; ‘--gene-type all’ returns all resistance and virulence bins). ReGAIN Module 1.0 currently supports the analysis of 30 bacterial species (Table 1), leveraging the latest AMRFinderPlus functionality. The organism non-specific pipeline analyzes genomes using the AMRFinder core gene catalog (Feldgarden *et al.* 2019) to detect common antibiotic resistance genes across species. AMRFinderPlus uses a combination of Hidden Markov Models and manually curated Basic Local Alignment Search Tool (BLAST) cutoffs to identify genes from a reference catalog of over 7000 resistance and virulence gene determinants (Feldgarden *et al.* 2021). Module 1.0 supports multi-threading using the built-in AMRFinderPlus parallelization framework and wraps all major AMRFinder options.

Dataset creation, Module 1.1, creates and curates all necessary data files for default downstream analysis within the ReGAIN pipeline, including a presence/absence matrix, a metadata file, and a concatenated AMRFinder results file. The presence/absence matrix encodes gene detection as binary values across the genomic population (1 = present, 0 = absent). This binary format is required for ReGAIN’s discrete Bayesian network analysis workflow. To ensure the dataset is suitable for analysis, Module 1.1 allows users to specify minimum and maximum gene occurrence thresholds. Genes that are in either very low or very high abundance should be excluded to reduce network noise. Because discrete Bayesian network analyses require features (e.g. genes) to exist in at least two states (i.e. any single feature must be both ‘present’ and ‘absent’ in the population), it is necessary to filter ubiquitously occurring genes from the analysis. Thus, for ReGAIN datasets, all genes identified across a genomic population must occur in both the ‘present’ and ‘absent’ states. To allow user-end flexibility, the ReGAIN workflow directs users to manually set minimum/maximum occurrence values; for *n* genomes, the maximum allowable occurrence for any given gene is *n—*1 (i.e. for 100 genomes, the maximum allowed occurrence for any single gene is 99, though a 95% maximum occurrence threshold is recommended). From here, users may choose to assess their genomes for resistance, virulence, or both gene types. Resistance genes include those associated with antibiotic and heavy metal resistance, as well as select stress response genes. The distribution of genes across the genomic dataset is reduced to a binary format to create a filtered presence/absence matrix based on the user-input minimum/maximum gene occurrence thresholds. The metadata file generated by Module 1.1 includes all identified genes and, for resistance determinants, functional annotations, such as resistance class (e.g. aminoglycoside, sulfonamide). Finally, a combined data file containing all AMRFinder results is generated. As resistance genes are often encoded both chromosomally and on extra-chromosomal genetic elements, important gene location information is retained. The final combined AMRFinder data file contains both contig/scaffold identification and nucleotide coordinates for each gene identified in Module 1 organized by source genome (Table 2). Because ReGAIN relies on AMRFinder/Plus annotations, which provides highly granular gene annotations that include specific allelic variants [e.g. *aac(6’)-Ib-cr* and *aac(6’)-Ib-cr5*], a supplementary ‘collapse-features’ script is provided for users who prefer to bin features into broader categories. This module recreates the output presence/absence matrix based on user-provided bins, which can be appended to the standard metadata.csv output file generated from ‘regain matrix’. Finally, Module 1.2 provides a ‘matrix-summary’ function, which calculates basic statistics of gene occurrence, including mean and median number of genes per genome (Table S1), as well as an exhaustive overview of unique and duplicate resistance profiles observed in the data (profile_groups.csv).

**Table 2:** Example of gene location data from combined AMRFinder results file using *Escherichia coli* as an example.

Contig Id	Start	Stop	Strands	Gene symbol
NZ_CP128944.1	1	720	+	*aph(3″)-Ib*
NZ_CP128944.1	723	1556	+	*aph(6)-Id*
NZ_CP128944.1	5266	6078	+	*sul2*
NZ_OZ040729.1	4382	45 084	‒	*tet(B)*
NZ_OZ040729.1	49 732	50 202	‒	*dfrA17*

ReGAIN Module 1.1 concatenates all AMRFinder (Feldgarden *et al.* 2021) results into a tab-separated file, maintaining the original organization and content. This table only highlights gene location data; the full table includes additional data, including gene description, gene class, percent identity, percent coverage, and accession of matching reference gene.

### 3.2 Overview of ReGAIN Module 2: Bayesian network structure learning

The ReGAIN Bayesian network structure learning module has been optimized for datasets of varying sizes. The module’s backend employs the bnlearn (Scutari 2010) and gRain (Højsgaard 2012) packages in R to generate and query Bayesian networks. Given the computational complexity associated with Bayesian network structure learning, two pipelines were designed based on the size and complexity of the input dataset to optimize performance and accuracy. For small-to-moderate size datasets (<100 genes), ReGAIN utilizes the gRain ‘querygrain’ function (regain bnS pipeline). This function is adept at handling smaller networks where exhaustive computations can be performed without prohibitive computational costs and full graph inference is manageable, thus allowing direct computation of conditional dependencies. For very large datasets (≥100 genes), ReGAIN leverages bnlearn’s ‘cpquery’, a function designed for large-scale Bayesian networks (regain bnL pipeline). As feature numbers (e.g. number of genes) increase, the computational requirements for direct calculations increase exponentially, quickly resulting in intractable computation costs. ‘cpquery’ addresses this issue by estimating conditional probabilities through Monte Carlo simulations (--cp-samples <int>, default = 10 000) (Scutari 2010, Ko *et al.* 2019, Warne *et al.* 2020).

Although ‘regain bnL’ provides a scalable alternative for querying Bayesian networks when full-graph inference is computational intractable, the two workflows use different inference strategies and should not be treated as directly interchangeable. For a given dataset, users should select either ‘bnS’ or ‘bnL’ as the network-querying framework and avoid combining or directly comparing results generated across both approaches. Importantly, we observed that, in rare cases, Monte Carlo estimations involving strongly linked gene pairs produced unstable and disproportionately large relative risk estimates. Across all ‘regain bnL’-generated example networks in this study, only 232 of 38 305 total gene-pair queries (0.61%) represented these unstable relative risk values (Table S2). These gene pairs were overwhelmingly represented by heavy metal resistance-associated genes known to strongly co-occur within operons (e.g. *mer, pco, sil* gene families). To prevent these fringe cases from confounding downstream interpretation, ‘regain bnL’ applies a cap to unusually large relative risk mean values. Associated confidence intervals and standard deviation are proportionally rescaled, while unadjusted values are retained and capped pairs are explicitly flagged in both the terminal output and results table. Together, these methods ensure that the ReGAIN pipeline remains computationally feasible, while maintaining a high degree of accuracy in the probabilistic assessment of gene co-occurrence. Although the Bayesian network analysis module is designed to interface with the results output from the data acquisition module, instructions on how to format externally prepared datasets are provided for users to run this module independently of Module 1 (Fig. S1).

**Figure 2:**
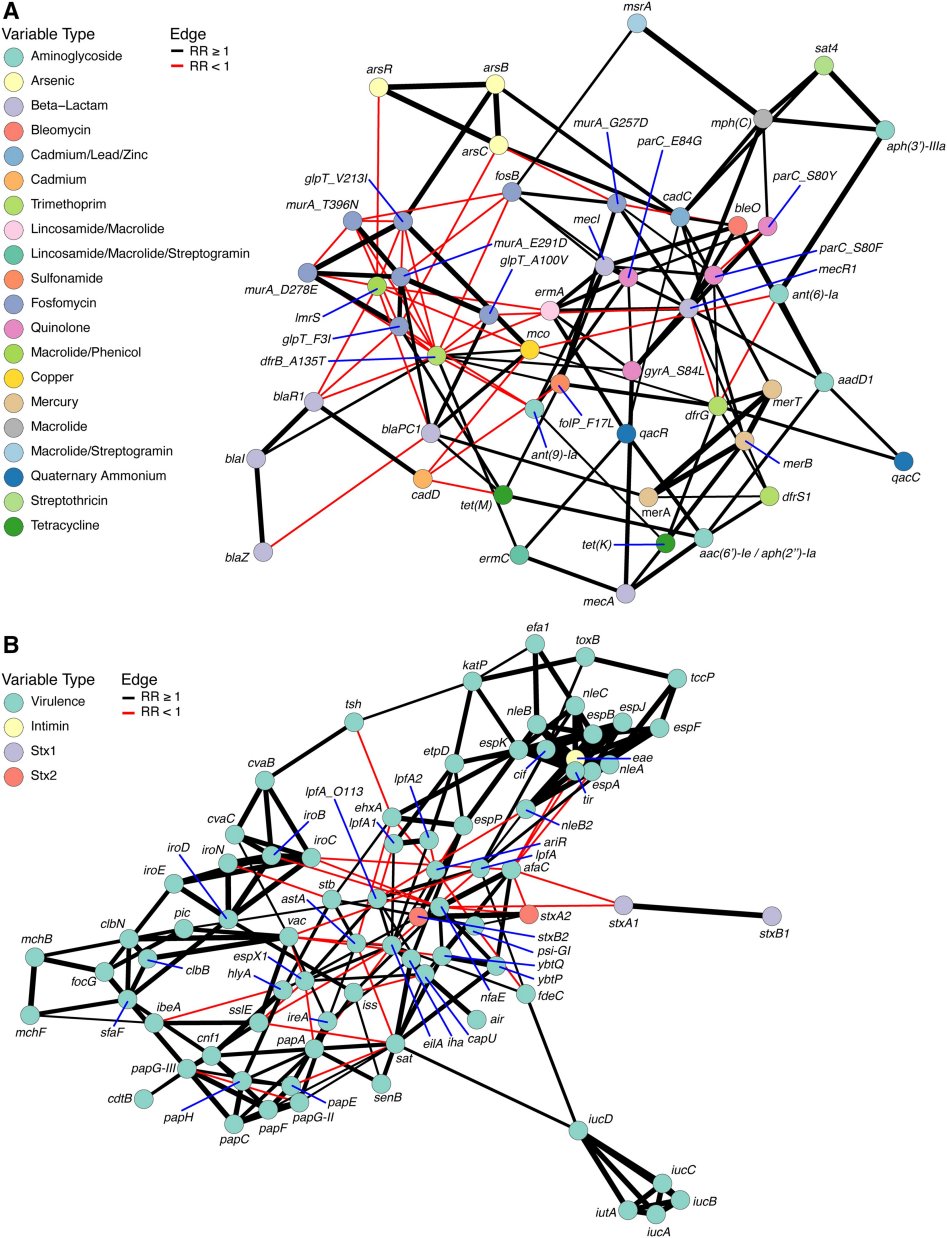
Example output of Bayesian network from ReGAIN Module 2. (A) Bayesian network created using 1000 *S. aureus* genomes representing 48 antibiotic resistance genes. (B) Bayesian network created using 1000 *E. coli* genomes representing 78 virulence genes. Nodes represent genes, while edges linking nodes indicate a probabilistic relationship between those genes. Analyses were performed using 500 bootstraps and 500 data resamples with replacement. Networks represent the best fit model. Nodes are color coded by gene type. Edge color: red = relative risk <1 (negative association); black = relative risk ≥1 (positive association). Edge width represents conditional probability confidence interval. PDF files were minimally edited in Illustrator for readability. Resistance genes in panel A were manually changed back to pre-special character sanitization to emphasize the utility of the PDF output for post-hoc figure customization.

Once executed, Module 2 employs a systematic approach to analyze the genomic data. First, Bayesian network structure learning using a Hill Climbing algorithm and Bayesian Dirichlet equivalent score is used to construct the network using a default imaginary sample size of 10 (--iss <int>). The combination of the two functions guides the algorithm in learning the most probable network structure given the data. A critical aspect of the Bayesian network workflow is the implementation of bootstrapping to resample data multiple times. Users are directed to use a minimum of 300 to 500 bootstraps to enhance the robustness of the network analysis. Following bootstrapping, the module applies a significance threshold of 0.5 to filter out weakly supported gene pairs from the network. ReGAIN Bayesian network analyses produce Directed Acyclic Graphs, such that gene pairs that would induce cycles in the graph (an example might be a gene pair with identical occurrence profiles or genes that represent strong reciprocal dependencies) are automatically pruned from the final network, with problematic variable pairs logged to the terminal. The refined network then undergoes further analysis through an additional resampling process. This step involves fitting multiple Bayesian networks to subsets of the data, which are randomly sampled with replacement, such that all values within the dataset have an equal probability of being selected one or more times. To enhance the statistical reliability of our example networks (Fig. 2, Fig. S2A–H, Table S2), each resampled dataset was used 500 times to fit the Bayesian network. Finally, statistical analyses are performed on the fitted networks. For each gene pair, mean conditional probability, mean relative risk, mean absolute risk difference, empirical confidence intervals, and standard deviation are calculated (Table 3). Conditional probability (Equation 1) is described as the probability of observing Gene A given the presence of Gene B, *P(A|B)*, while relative risk (Equation 2) is the ratio of the conditional probability of observing Gene A given Gene B to the conditional probability of observing Gene A in the absence of gene B, *P(A|B)/P(A|¬B)* (i.e. the probability of co-occurrence divided by the probability of single occurrence). Whereas conditional probability is expressed on a scale of 0 to 1 (e.g. a conditional probability of 0.65 indicates a 65% conditional probability of gene co-occurrence), relative risk offers a more descriptive scale. For instance, a relative risk of 1 suggests that Gene A and Gene B are likely independent of each other, A ⫫ B. A value greater than 1 suggests that it is more likely to observe Gene A in the presence of Gene B, as *P(A|B) > P(A|¬B).* Conversely, a value less than 1 indicates that Gene A is more likely to be observed independently of Gene B, as *P(A|B) < P(A|¬B)*.

**Table 3:** Example Module 2 output of conditional probability and relative risk results from *Escherichia coli*.

Gene_1	Gene_2	CPRMean	CPR SD	CPR CI (low)	CPR CI (high)	RR Mean	RR SD	RR CI (low)	RR CI (high)	ARD Mean	ARD SD	ARD CI (low)	ARD CI (high)
*aph(3″)-Ib*	*aph(6)-Id*	0.991	0.006	0.990	0.991	138.4	209.5	120.0	156.8	0.980	0.008	0.979	0.981
*aph(3″)-Ib*	*sul2*	0.862	0.02	0.860	0.864	6.9	0.839	6.8	7.0	0.735	0.02	0.733	0.737
*sul2*	*aph(6)-Id*	0.768	0.02	0.766	0.770	9.7	1.5	9.6	9.9	0.687	0.03	0.685	0.689

CPR: conditional probability; SD: standard deviation; CI: confidence interval; RR: relative risk, ARD: absolute risk difference. Here, streptomycin resistance genes, *aph(6)-Id* and *aph(3″)-Ib* and the sulfonamide resistance gene, *sul2*, were chosen as example genes to serve as proof of concept for ReGAIN’s statistical approach, as they have been observed to co-occur in Enterobacteriaceae (Indugu *et al.* 2020, Ikhimiukor *et al.* 2022, Tang *et al.* 2022). All three genes exhibit strong probabilities of co-occurrence (ARD > 0.5, RR > 1).

Equation 1. Conditional probability. The probability of observing ‘Gene A’ given the presence of ‘Gene B’.


$$P(A|B)=\frac{P(B|A)\cdot P(A)}{P(B)}$$

Equation 2. Relative risk. The ratio of the conditional probability of observing ‘Gene A’ given the presence of ‘Gene B’ to the conditional probability of observing ‘Gene A’ in the absence of ‘Gene B’. This calculation adjusts for single occurrence of Gene A in the dataset, offering a more in-depth understanding of the probability and rate of co-occurrence.


$$RR=\frac{P(A|B)}{P(A|\neg B)}$$

Finally, absolute risk difference is calculated for all gene pairs. Absolute risk difference is defined as the conditional probability of observing Gene A given Gene B, *P(A|B)* (co-occurrence) minus the conditional probability of observing Gene A by itself, *P(A|¬B)* (single occurrence). Relative risk and absolute risk difference are both calculated to provide complementary summaries of gene co-occurrence. While relative risk describes the proportional change in the probability of co-occurrence, absolute risk difference describes the overall probability of observing co-occurrence adjusted for the probability of single occurrence.

For bnS analyses, baseline risk of outcome is additionally calculated [i.e. the prevalence of an outcome variable (Gene B) in the dataset; a baseline risk of 0.1 indicates that Gene B is present in 10% of the input genomic population]. Aside from a table containing statistical summaries, conditional probability, relative risk, and absolute risk difference, the output of the Bayesian network analysis module includes the refined network as an interactive HTML file (Fig. S2A–H) and as a static PDF file, with the network layout utilizing the Fruchterman-Reingold force-directed layout algorithm. For reproducible layouts, ReGAIN uses a set seed; this value may be overridden using the optional network visualization module (--seed <int>, default = 42).

ReGAIN’s core modules currently support analysis of antibiotic and heavy metal resistance genes, as well as select stress determinants (Fig. 2A) and virulence genes (Fig. 2B) using the organism-specific workflow. For both Bayesian network structure learning pipelines, users may submit a table of variable pairs to blacklist from the analysis (--blacklist <file.csv>). Blacklists should be formatted as a two-column CSV file with no headers and variable pairs should be explicitly bi-directional (i.e. Gene A, Gene B and Gene B, Gene A). Module 2.1 includes a ‘network-analysis’ function for performing sensitivity analyses between two ReGAIN results sets. This module compares shared and unique associations and quantifies changes in conditional probability, relative risk, and absolute risk difference between genomic datasets. This allows users to assess whether inferred gene-gene associations are robust across different genomic populations. Importantly, special characters (e.g. parenthesis, hyphens, quotes, etc.) must be excluded from gene names prior to submission to ReGAIN Module 2, as the Bayesian network backend cannot parse special characters. For ease of analysis, this transformation is automated during dataset creation. Quotation marks are replaced by ‘p’ (i.e. ‘prime’); parenthesis are removed; periods, slashes, and hyphens are replaced by ‘_’: *aph(3″)-Ib* becomes *aph3pp_Ib*, *mcr-1.1* becomes *mcr_1_1*. If desired, this process can be overridden by using the ‘--keep-gene-names’ flag; however, this option should be used with caution, as variables with special characters will be ignored during structure learning.

To supplement and further analyze probabilistic relationships of gene co-occurrence, two post-hoc analyses are performed. Bidirectional probability score (BDPS) (Equation 3) describes the directional strength of the conditional probabilities of a gene pair. As conditional probability, *P(A|B)*, is dependent on the presence or absence of Gene B, the reciprocal conditional probability is not often equal, *P(A|B) ≠ P(B|A)* [e.g. knowing that it is raining (B) changes the probability that grass is wet (A), but knowing that grass is wet doesn’t change the probability of rain in the same way]. Therefore, a better assessment of the overall strength of the relationship can be achieved by taking the ratio of these two values. If BDPS = 1, equal bidirectional strength can be inferred. If BDPS >1, the probability of observing Gene A given Gene B is stronger, *P(A|B) > P(B|A)*. Conversely, if BDPS <1, the probability of observing Gene B given Gene A is stronger, *P(A|B) < P(B|A)*.

Equation 3. Bidirectional probability score. The ratio of the conditional probability of observing ‘Gene A’ given the presence of ‘Gene B’ to the conditional probability of observing ‘Gene B’ given the presence of ‘Gene A’.


$$\mathrm{BDPS}=\frac{P(A|B)}{P(B|A)}$$

The second post-hoc analysis is fold change, which is calculated using relative risk scores (Equation 4). Fold change is interpreted similarly to BDPS. A fold change >1 indicates that the relative risk ratio of Gene A given Gene B is stronger, *RR(A|B)* > *RR(B|A)*, while a fold change <1 indicates that the relative risk ratio of Gene A given Gene B is weaker, *RR(A|B)* < *RR(B|A)*. Importantly, a fold change value of 1 may indicate either equal bidirectional probability or variable independence, dependent on the constituent relative risk values. To expand on this, if *RR(A|B)* and *RR(B|A)* both exhibit a value of 3, indicating strong equal bidirectional strength, the fold change would be 1; however, if *RR(A|B)* and *RR(B|A)* both exhibit a relative risk of 1, indicating a neutral relationship between the gene pair, the fold change would also be 1. Because of this, it is important to use these post-hoc analyses in context of the underlying conditional probability and relative risk values of each gene pair. Taken together, ReGAIN’s Bayesian network structure learning module offers valuable insight into both the probability and directional strength of gene co-occurrence.

Equation 4. Fold change. The ratio of the relative risk of ‘Gene A’ to ‘Gene B’ to the relative risk of ‘Gene B’ to ‘Gene A’.


$$FC=\frac{\left(\frac{P(A|B)}{P(A|\neg B)}\right)}{\left(\frac{P(B|A)}{P(B|\neg A}\right)}$$

### 3.3 Impact of population size on network structure

**Figure 3:**
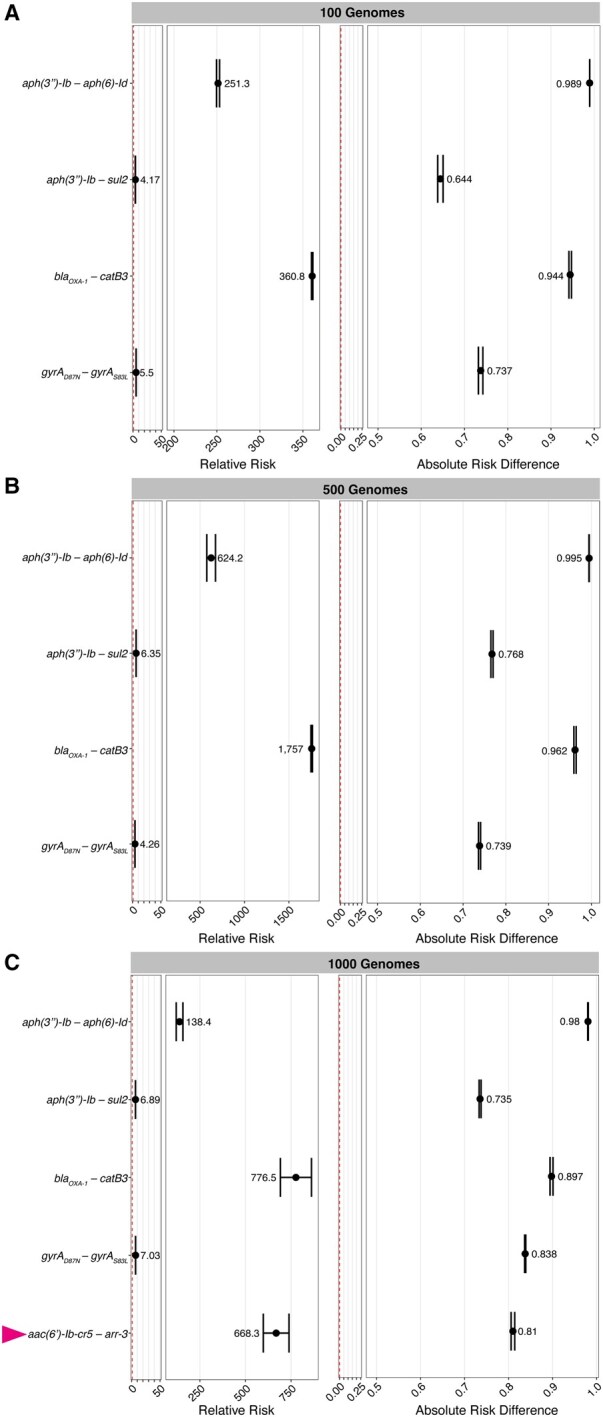
Forest plots comparing the effect of genomic population size on inferred gene-pair probability metrics in *E. coli*. Mean relative risk and absolute risk difference values are shown for select gene pairs derived from Bayesian networks generated using (A) 100, (B) 500, or (C) 1000 *E. coli* genomes. The red dotted line represents ‘no correlation’ values (relative risk = 1, absolute risk difference = 0). The magenta arrow in panel (C) highlights a gene pair absent from the 100- and 500-genome datasets due to low occurrence and captured only in the 1000-genome dataset. Error bars represent confidence intervals.

To determine how genomic population size affects ReGAIN network output, we generated three Bayesian network result sets using randomly selected populations of 100, 500, and 1000 publicly available *E. coli* genomes. We then used ‘regain network-analysis’ to perform non-redundant pairwise comparisons across population sizes (Table S3). Across comparisons, conditional probability and absolute risk difference remained relatively stable, with mean changes of 0.009 for conditional probability and 0.018 for absolute risk difference, respectively (Table S3). In contrast, relative risk showed greater variability across population sizes (mean Δ RR: 15.57), consistent with the sensitivity of ratio-based metrics to changes in low-frequency features. To further evaluate population-size effects at the individual gene-pair level, we examined several known co-occurring resistance determinants, including *aph(3″)-Ib* and *aph(6)-Id, aph(3″)-Ib* and *sul2*, *bla_OXA-1_*, and *catB3*, and *gyrA_D87N_* and *gyrA_S83L_* (Bring Horvath *et al.* 2025) (Fig. 3). Absolute risk difference values remained comparatively stable across these gene pairs (overall mean Δ ARD: −0.023), whereas relative risk values showed greater pair-specific variability (overall mean Δ RR: −51.17), as expected for a ratio-based metric.

Because relative risk is calculated from the ratio of conditional probabilities (Equation 2), small changes in the denominator can produce disproportionately large apparent shifts, particularly when the probability of observing gene A in the absence of gene B, *P(A|¬B)*, is substantially smaller than the probability of observing gene co-occurrence, *P(A|B)*. Accordingly, relative risk should be interpreted alongside the underlying conditional probability and absolute risk difference values.

The largest practical difference among population sizes was not a broad restructuring of the network, but rather the number of gene pairs retained for downstream analysis. Because ReGAIN applies low- and high-occurrence thresholds to filter features prior to network construction, smaller genomic populations are less likely to retain lower-frequency determinants. For example, the association between *aac(6′)-Ib-cr5* (aminoglycoside/quinolone) and *arr-3* (rifamycin) was excluded from the 100- and 500-genome datasets because *arr-3* did not meet the minimum-occurrence threshold; however, this association was retained in the 1000-genome dataset. Together, these results suggest that overall network structure is reproducible across population sizes for more commonly occurring genes, while larger genomic populations improve detection of lower-frequency resistance determinants.

### 3.4 Impact of genome similarity on network structure

To evaluate the effect of near-clonal genome relatedness on ReGAIN network structure, we developed the ‘regain genome-similarity’ module. This module allows users to perform pairwise genome similarity analyses using either average nucleotide identity (ANI) or Mash distance, cluster genomes using default or user-defined similarity thresholds, and identify representative genomes from highly similar genome clusters for downstream investigation. For this analysis, we used a random starting cohort of 500 publicly available *E. coli* genomes and generated three Bayesian networks: an unadjusted network containing all 500 genomes, a reduced network retaining one representative genome per cluster at a 99.9% ANI threshold (n = 349), and a reduced network retaining one representative genome per cluster at a 99.5% ANI threshold (n = 159). Overall network metrics remained largely stable following genome-similarity-based population reduction (Table S4). Across comparisons, conditional probability and absolute risk difference showed minimal change, with average differences of 0.0073 and 0.013, respectively. Relative risk again showed greater sensitivity to population reduction, with an average change of 9.46, consistent with the behavior of relative risk as a ratio-based metric. As observed in the population-size analysis (Table S3), the largest practical difference among networks was the number of gene pairs retained for downstream analysis, again attributable to the low- and high-occurrence thresholds applied prior to network construction. Reducing the input population altered which genes meet inclusion criteria, particularly for lower-frequency determinants.

**Figure 4:**
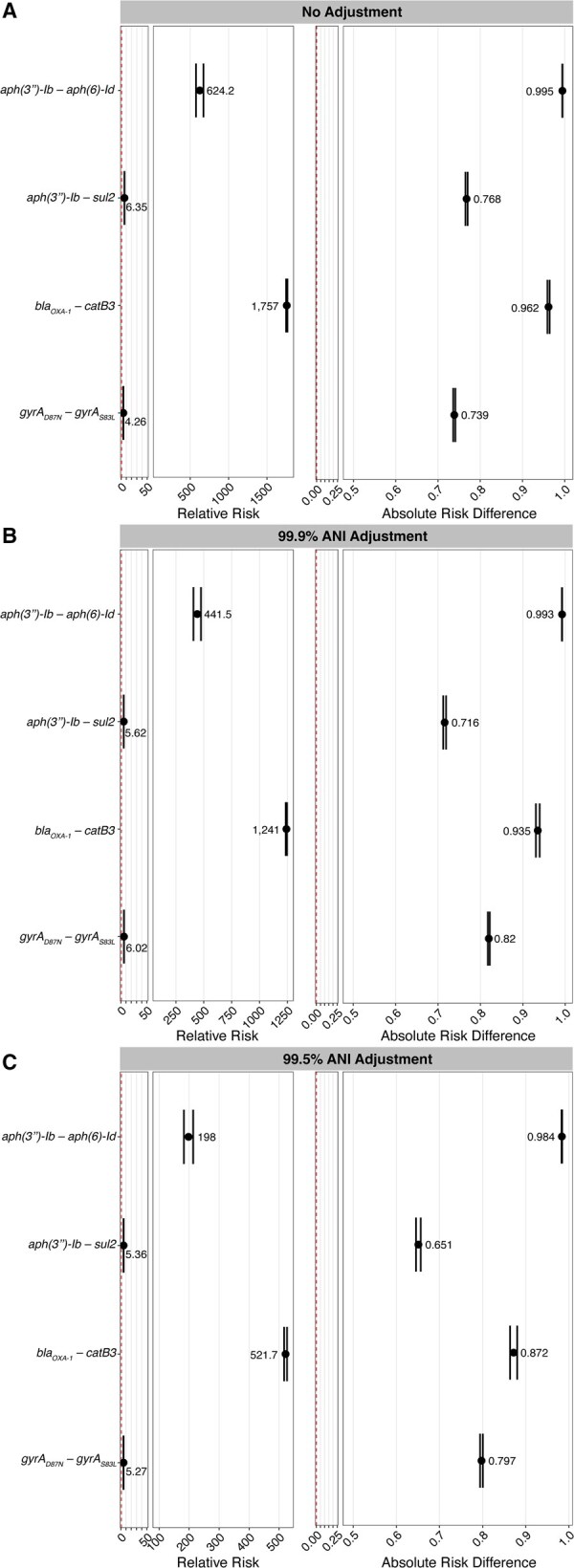
Forest plots comparing the effect of genome-similarity-based population reduction on specific gene-pair probability metrics in *E. coli*. Mean relative risk and absolute risk difference values for select gene pairs derived from Bayesian networks generated using (A) 500 *E. coli* genomes with no genome similarity-based adjustments, (B) 349 genomes using an average nucleotide identity (ANI) threshold of 99.9% similarity, and (C) 159 genomes using an ANI threshold of 99.5%. The red dotted line represents ‘no correlation’ values (relative risk = 1, absolute risk difference = 0). Error bars represent confidence intervals.

At the individual gene-pair level, the underlying conditional probability values remained similar between the unadjusted and genome-similarity-reduced populations (Fig. 4). Higher relative risk values were generally observed in the unadjusted population, with the exception of *gyrA_D87N_–gyrA_S83L_*. We interpret this pattern cautiously, as higher relative risk values in the unadjusted network likely reflect real population structure rather than technical inflation. Highly related genomes may represent biologically meaningful clonal expansion, including repeated observations of linked resistance determinants within similar genomic backgrounds. Conversely, aggressive reduction of highly similar genomes may decrease the power to detect biologically relevant associations, particularly for resistance determinants encoded on extra-chromosomal genetic elements, such as plasmids, that are not strictly inherited vertically. Together, these results suggest that near-clonal population structure can influence the number and magnitude of detected associations but do not substantially alter the dominant network relationships within this test dataset. Thus, the default ReGAIN workflow preserves the full input population to maximize detection of gene-gene co-occurrence patterns, while ‘regain genome-similarity’ provides users with an optional tool to identify highly similar genome clusters when appropriate for the study design.

### 3.5 Overview of ReGAIN Curate: a flexible and scalable data acquisition module for additional genomic queries

In addition to ReGAIN Module 1, which identifies genes using AMRFinder/Plus, ReGAIN Curate was developed to allow users to query their own genes of interest across a genomic population and curate datasets tailored to specific research goals. While AMRFinderPlus relies on a large reference gene database, certain genes may be missed. For example, when analyzing the multidrug-resistant extraintestinal pathogenic *E. coli* strain U15A (Forde *et al.* 2019) (NCBI Accession: NZ_CP035720.1), AMRFinderPlus failed to annotate the chromate efflux gene *chrA* (Zhang *et al.* 2016) and the macrolide efflux gene *mrx(A)* (Poole *et al.* 2006), both of which are often constituents of a large, plasmid-encoded resistance island (Forde *et al.* 2019, Bring Horvath *et al.* 2025). ReGAIN Curate was developed to address such limitations.

ReGAIN Curate uses a heuristic BLAST-based approach to identify user-defined reference genes in a genomic population. First, a nucleotide database is constructed using NCBI BLAST+ (Camacho *et al.* 2009) and performs translated BLAST (tblastn) queries, expecting protein sequences by default. Users may also submit nucleotide sequences using the optional ‘--nucleotide-query’ option. Internally defined thresholds use a default minimum of 90% sequence identity and 75% query coverage to report a positive match, balancing sensitivity and specificity. These values may be overridden using the ‘--perc’ and/or ‘--cov’ options. Users provide three inputs: a genomic population, a set of reference genes, and minimum/maximum gene occurrence thresholds (similar to Module 1.1). ReGAIN Curate generates a binary presence/absence matrix and corresponding metadata file, both compatible with ReGAIN Module 2 for downstream analysis. When multiple hits occur, only the strongest BLAST match is retained in the binary output matrix.

ReGAIN Curate is a standalone module that creates BLAST databases, performs gene queries, and generates filtered datasets in a single, streamlined pipeline. If additional validation is desired, users may extract aligned nucleotide sequences using the ReGAIN Extract module. This module generates a multi-FASTA file, with each sequence annotated with the genome of origin and the corresponding query identifier. In the event users wish to combine results from ReGAIN Curate with those obtained using ReGAIN Module 1, we developed ReGAIN Combine, a simple utility program designed to combine data matrix and metadata outputs from both ReGAIN Module 1 and ReGAIN Curate. This combined dataset can then be directly submitted to Module 2.

Because ReGAIN Module 1 is based on the large reference library of AMRFinderPlus, it can be computationally time consuming. While it offers robust gene detection, ReGAIN Curate provides a faster alternative for targeted analysis using smaller, user-defined gene sets. As with Module 1, ReGAIN Curate supports parallel processing. To assess its performance, we queried 20 genes from the Ec17R resistance island encoded by plasmid pU15A_A (NZ_CP035721.1) (Forde *et al.* 2019) (Table S5) against a dataset of 500 *E. coli* genomes, representing 10 000 total queries. This cohort included both antibiotic and heavy metal resistance genes and associated mobile genetic elements present within the island. This gene set was chosen as it represents a biologically relevant mobile resistance region containing determinants associated with resistance to clinically used antibiotics. The resistance genes from Ec17R included *aph(6)-Id*, *aph(3″)-Ib*, and *aadA5* (streptomycin resistance); *sul1* and *sul2* (sulfonamide resistance); *eamA* (small molecule efflux); *tet(A)* (tetracycline resistance); *chrA* (chromate efflux); *aac(3)-IId* (gentamicin resistance); *dfrA17* (trimethoprim resistance); *mph(A)*, *mrx(A)*, and *mphR(A)* (macrolide resistance); *qacEdelta1* (quaternary ammonium efflux); and *tmrB* (putative tunicamycin resistance), as well as several TEs present within the island (Forde *et al.* 2019, Bring Horvath *et al.* 2025) (Table S5). Runtime was benchmarked using one, two, four, and eight dedicated cores. The analytical times are as follows [minutes ± standard deviation (SD)]: one CPU, 16.3 ± 0.12; two CPUS, 8.71 ± 0.07; four CPUs, 4.67 ± 0.01; eight CPUs, 2.72 ± 0.03. This inverse relationship between core count and runtime highlights the scalability of the ReGAIN Curate pipeline (Fig. S3). When the number of reference genes was increased from 20 to 123 as a stress test (representing all predicted coding sequences encoded by pU15A_A; 61 500 queries), analysis time remained reasonable at approximately 35 minutes using eight cores.

To evaluate the accuracy of ReGAIN Curate, we compared its performance to that of AMRFinderPlus across 28 resistance and virulence genes in 500 *E. coli* genomes. Reference sequences (Table S6) were obtained from *E. coli* strain U15A and queried using the ReGAIN Curate pipeline. AMRFinderPlus results served as the control and ReGAIN Curate hits were assessed as percent accuracy (Table S7). Of the 28 genes tested, 22 had accuracies between 98.8% and 103.4%, with values >100% indicating additional calls by AMRFinderPlus (Table S7). *tet(A)* (104.2%) and *aadA5* (109.0%) were modest ReGAIN Curate under-calls, while *iutA* (116.3%) and *sat* (125.0%) represent larger outliers. *aac(3)-IId* and *emrD* exhibited accuracies of 76.8% and 44.6%, respectively, indicating that ReGAIN Curate was calling more gene matches than AMRFinderPlus. Further investigation revealed that *emrD*, a member of the Major Facilitator Superfamily (MFS), returned numerous hits likely representing closely related genes. When we increased the inclusion thresholds to 99% identity and 99% coverage, the accuracy of *emrD* gene calls increased from 44.6% to 110.2%, suggesting that AMRFinderPlus recognizes multiple *emrD* sequence variants not captured by a single reference query. Similarly, *aac(3)-IId* belongs to a large aminoglycoside *N*-acetyltransferase family (Stogios *et al.* 2022); the additional 23.2% of *aac(3)-IId* hits identified by ReGAIN Curate were reclassified as *aac(3)-IIe* (Table S7), a closely related sequence variant, upon manual annotation using the NCBI nr_cluster_seq database. In another case, ReGAIN Curate returned two additional hits for *qacEdelta1* that aligned to *qacE*, a closely related homolog. Lowering the query coverage threshold to 50% helped recover several under-reported genes but also introduced a slight increase in over-calling, emphasizing the need to balance sensitivity and specificity based on research goals. Overall, ReGAIN Curate displayed high accuracy in gene calling, emphasizing its utility in supplementing ReGAIN Module 1, especially in identifying genes that are absent from the AMRFinder reference library. Together, the ReGAIN Curate module provides a flexible and scalable pipeline for preparing datasets compatible with downstream statistical analysis. Moreover, the use of stringent internal BLAST thresholds, coupled with the ability to extract aligned sequences for validation via ReGAIN Extract, supports the accurate curation of user-defined genomic datasets.

### 3.5 Additional statistical analyses

**Figure 5:**
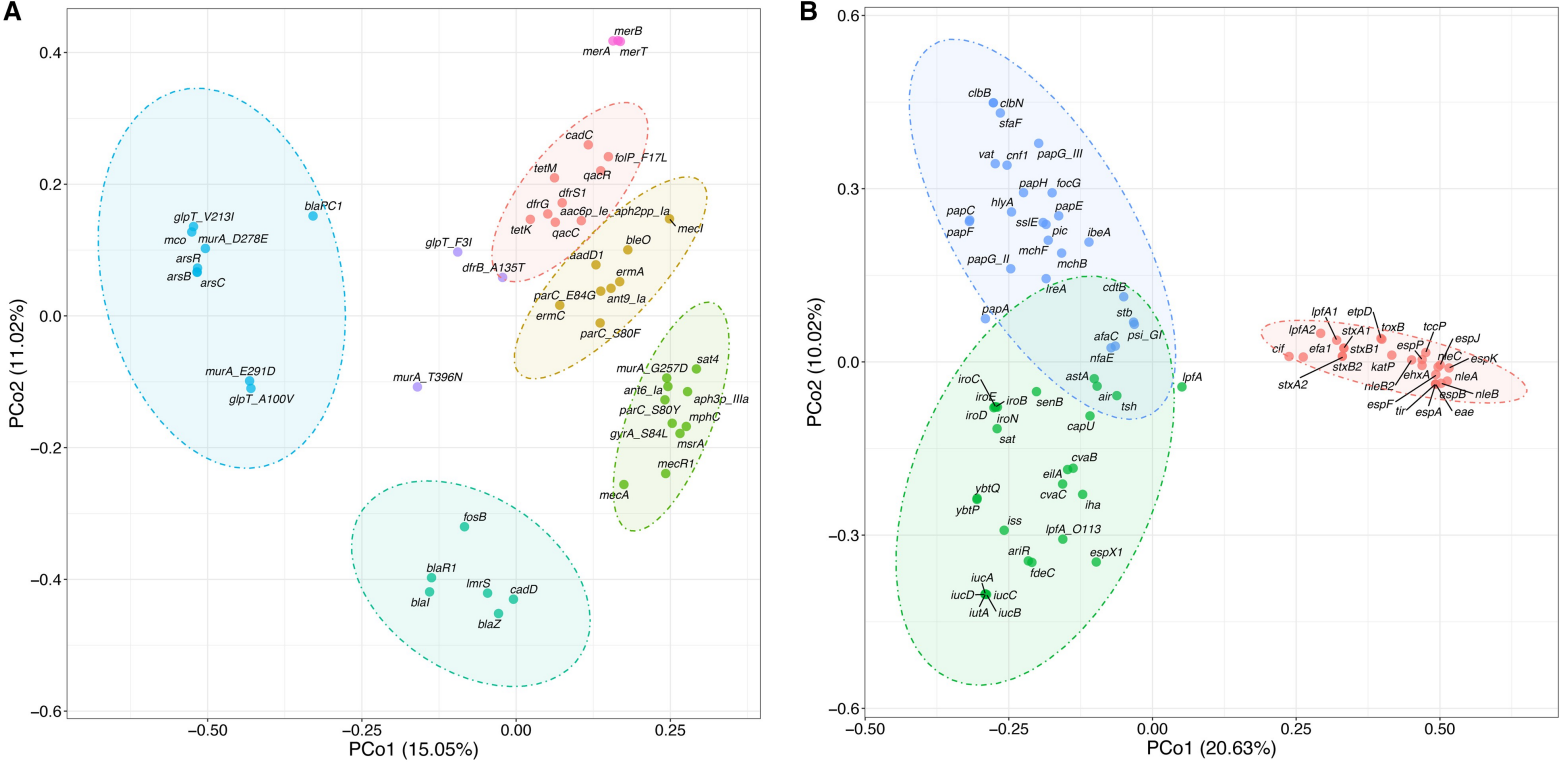
Example principal coordinate analysis (PCoA) output from the ReGAIN Additional Analyses Module. (A) PCoA representing similarities/dissimilarities in occurrence of 48 resistance genes identified in a *S. aureus* genomic population. (B) PCoA representing similarities/dissimilarities in occurrence of 78 virulence genes identified in an *E. coli* genomic population. PCoA plot was generated using the Jaccard measure of similarity. Ellipses represent 95% confidence, with --k 0 (auto) specified. For both *S. aureus* and *E. coli*, 1000 genomes were assessed for resistance or virulence genes. Figures were minimally edited in Illustrator for readability. Unlike Fig. 2A, gene names were not modified back to their pre-sanitized form for use as an example of an output ‘regain MVA’ plot.

In addition to Bayesian network structure learning, ReGAIN offers a multivariate analysis module (MVA) for quick exploration of datasets (Fig. 5). While Bayesian networks can reveal probabilistic relationships and dependencies between variables, MVAs can quickly provide insight into the patterns and structures of variables within a dataset. Further, the use of a k-means clustering algorithm allows for the categorization of data into clusters based on similarity measures, which may assist in identifying distinct groups or outliers. This can be an important step in understanding the spread and development of resistance patterns, especially as a dataset grows over time and becomes more complex.

For this module, an ordination-and-clustering workflow to explore global structure on ReGAIN matrices was implemented. Input is the ‘regain matrix’-generated data matrix file. Logical/factor columns are coerced into numeric and missing values are set to 0 as a safety fallback. Pairwise dissimilarities are computed using user-selectable metrics (Jaccard, Bray, Manhattan, etc.). Additional modes supported by the vegan (Oksanen *et al.* 2022) R package are also supported. Principal coordinate analysis (PCoA) is then applied to the distance matrix; percent variance explained by the first two axes is reported. The pipeline automatically applies a PCoA correction (default: auto; Cailliez when negative eigenvalues are detected, otherwise none), with explicit lingoes/cailliez/none options available, and reports corrected relative eigenvalue percentages when available. Clustering is performed by k-means on the first two PCoA axes. Users may fix *k* or request auto-selection (--k 0), which scans *k *= 2…10 and chooses the value that maximizes mean silhouette width (clusters with <3 points are skipped during selection to avoid degenerate solutions). For visualization, samples are plotted in the PCoA plane with optional labels. When clusters contain ≥3 distinct points and have positive-definite covariance, normal-theory confidence ellipses are overlaid (default = 95%) (Murdoch and Chow 1996); otherwise ellipses are omitted and a diagnostic message is generated. A fixed random seed (default = 42) ensures reproducibility. Outputs include publication-quality figures (MVA.png, MVA.pdf), tabulated 2D coordinates with cluster assignments (MVA_coordinates.csv), and—optionally—the full distance matrix (--save-dist). The workflow is intended for exploratory structure discovery; choices of distance, transformation, and *k* should be interpreted in the context of study design and data type. Using the same example *S. aureus* and *E. coli* datasets used previously (Fig. 2), PCoA plots were generated to explore resistance genes in *S. aureus* (Fig. 5A) and virulence genes in *E. coli* (Fig. 5B).

## 4 Discussion

Rising rates of multidrug resistance represent a global health threat that is unlikely to be resolved any time soon (Prestinaci *et al.* 2015, Munita and Arias 2016, Manyi-Loh *et al.* 2018, Diekema *et al.* 2019, Sharma *et al.* 2022). However, when applied to well-curated genomic datasets, probabilistic statistical models can provide valuable insights into trends and patterns of resistance gene co-occurrence. The ReGAIN bioinformatic pipeline offers a reproducible method for assessing resistance gene co-occurrence. Because the transfer of resistance determinants is often mediated by TEs or plasmid-encoded loci, using ReGAIN alongside tools such as TnCentral (Ross *et al.* 2021), ISEScan (Xie and Tang 2017), or PlasmidFinder (Carattoli *et al.* 2014) may allow for a more comprehensive annotation of potential resistance islands and their associated mobile genetic elements. To support this type of analysis, ReGAIN generates a combined results file that lists all identified gene determinants across all queried genomes, along with their corresponding contig or scaffold IDs and nucleotide coordinates. Additionally, ‘regain matrix-summary’ identifies and reports all resistance profiles (i.e. empirical patterns of gene occurrence) identified in the genomic population. From an exploratory perspective, ReGAIN can aid in identifying previously unrecognized gene pairs that may act synergistically or contribute to resistance gene co-selection under clinical or environmental pressures. With this in mind, identification of heavy metal resistance genes is included in the ReGAIN workflow, as there is evidence of synergy or co-selection between heavy metal resistance genes and ARGs in bacterial pathogens (Akinbowale *et al.* 2007, Zhang *et al.* 2016, Yang *et al.* 2020). Importantly, while the ReGAIN Bayesian network structure learning module can be used to identify positive probabilistic relationships of resistance gene co-occurrence, it may also be used to find negative relationships (i.e. genes that may be mutually exclusive). These gene pairs would exhibit very low conditional probabilities and relative risk values <1 and would be readily identifiable in the ReGAIN results file generated from Module 2 and in the output network (red edges).

While Bayesian network structure learning is a powerful statistical approach for identifying and mapping co-occurrence patterns among resistance genes, it is important to recognize its limitations. Sample size may influence results, especially if the sample size is very small, resulting in the loss of potentially important features due to low abundance. However, when applied to well-curated genomic datasets, ReGAIN may offer predictive insights into the emergence and spread of multidrug resistance over time. Similarly, while AMRFinderPlus provides a robust method for identifying resistance and virulence genes, it may overlook important genes if they are absent from the reference database. To address this limitation and enhance user flexibility, ReGAIN offers the Curate pipeline, which allows users to query custom gene sets across genomic populations to create datasets tailored to specific research questions. It is important to note, however, that ReGAIN Curate may under- or over-report gene presence in a genomic population. This can be mitigated by querying multiple sequence variants of a target gene and comparing their presence/absence profiles prior to final dataset creation (i.e. dynamic querying). AMRFinder, for example, relies on a highly curated reference gene database (Feldgarden *et al.* 2019, 2021) that often includes multiple representative sequence variants per gene to improve gene-calling accuracy. When querying genes with high sequence similarity to other gene family members, users can increase the stringency of BLAST searches by adjusting the percent identity ‘--perc’ and query coverage ‘--cov’ parameters to reduce false positives. Additionally, understanding the biological context of target genes is critical for curating accurate datasets. To support this, the ReGAIN Extract module allows users to retrieve aligned sequences identified by ReGAIN Curate, facilitating further validation and/or functional annotation prior to analysis.

Taken together, the ReGAIN core and Curate pipelines offer researchers a reproducible and modular framework for assessing resistance and virulence gene co-occurrence across bacterial genomes. ReGAIN represents a novel open source bioinformatic platform specifically designed to enable probabilistic co-occurrence analysis of resistance and virulence genes, providing a powerful tool for exploring gene networks in bacterial pathogens.

## Supplementary Material

btag505_Supplementary_Data

## Data Availability

ReGAIN is available through Bioconda (https://anaconda.org/bioconda/regain-cli) or as a standalone installation (https://github.com/ERBringHorvath/regain_CLI). All ReGAIN analyses are currently performed using Python v3.11.5 and R v4.4.1. Please read the ReGAIN documentation for information on downloading and running the program. All genomes used were downloaded from the National Center for Biotechnology Information (NCBI) database. Large supplementary tables and results data from the ESKAPEE pathogen example network analyses can be downloaded from https://figshare.com/articles/dataset/ReGAIN_command_line_software_and_supplemental_figures_/28959431.
