# Supplementary material for: ReGAIN: a bioinformatics platform for assessing probabilistic co-occurrence between resistance genes in bacterial pathogens
Source: Bioinformatics. 2026 Jul 9;42(7):btag505. doi: 10.1093/bioinformatics/btag505 (PMC13401475; doi:10.1093/bioinformatics/btag505)
Supplement: btag505_Supplementary_Data [file btag505_supplementary_data.docx]

**Supplementary Information**

**ReGAIN: A bioinformatics pipeline for assessing probabilistic co-occurrence between resistance genes in bacterial pathogens**

**Author Information**

Elijah R. Bring Horvath^1^, Mathew G. Stein^2^, Matthew A. Mulvey^3,4^, Edgar J. Hernandez^5,*^, Jaclyn M. Winter^1,*^

^1^ Department of Pharmacology and Toxicology, University of Utah, Salt Lake City, Utah, 84112, United States

^2^ Independent researcher, Boise, Idaho, 83709, United States

^3^ School of Biological Sciences, University of Utah, Salt Lake City, Utah 84112, United States

^4^ Henry Erying Center for Cell & Genome Science, University of Utah, Salt Lake City, Utah, 84112, United States

^5^ Department of Biomedical Informatics, University of Utah, Salt Lake City, Utah, 84108, United States

* To whom correspondence should be addressed. Tel: [+1 801-581-6353]; Email: [jaclyn.winter@utah.edu]. Correspondence may also be addressed to Edgar J. Hernandez. Tel: [+1 801-587-5236]; Email: [edgarh@genetics.utah.edu].

**Table of Contents**

**Supplementary Figures**

Figure S1: Required format of externally prepared data using ReGAIN **S5**

Figure S2 A–H: Bayesian Networks generated from example genomic datasets **S6**

Figure S3: ReGAIN Curate performance **S10**

**Supplementary Tables**

Table S1: Example summary output of ‘regain matrix-summary’ **S3**

Table S2: Overview of the example ESKAPEE datasets used for proof-of-concept Bayesian

network structure learning **S4**

Table S3: Comparison of Bayesian network results across different genomic population sizes **S7**

Table S4: Comparison of ReGAIN Bayesian network results between populations adjusted

for genomic similarity **S8**

Table S5: Reference genes used in ReGAIN Curate test dataset **S9**

Table S6: Genes used to assess accuracy of ReGAIN Curate gene calling **S11**

Table S7: Accuracy of gene calling using the ReGAIN Curate pipeline **S12**

Supplementary references **S13**

**Table S1.** Example summary output of ‘regain matrix-summary’. This table reports metrics from the 1000-genome *E. coli* example dataset. Features comprising each profile group, as well as the genomes encoding them can be found in the ‘profile_groups.csv’ output file. Empirical counts and percent-of-total values for each feature can be found in ‘feature_counts.csv’ output file. Both files are generated by default during the ‘regain matrix-summary’ analysis.

**Table S2**. Overview of the example ESKAPEE datasets used for proof-of-concept Bayesian network structure learning. Features = genes.

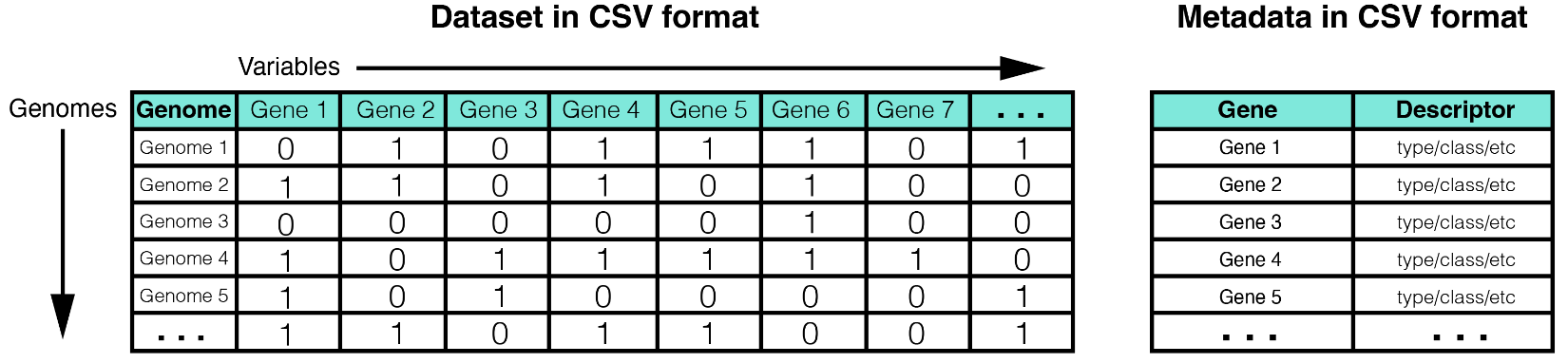


**Figure S1**. Required format of externally prepared data. The data matrix must be in presence/absence format, where ‘1’ = present and ‘0’ = absent. Rows and columns must be labeled by genome and gene, respectively. Each variable must have two states (i.e., both '1' and '0'). Including variables that have only one state, such as a gene that is present in every genome will result in the failure of the Bayesian network analysis. Variables may only contain alphanumeric characters and underscores (Do NOT include quotation marks, periods, forward or reverse slash marks, hyphens, parentheses). Addition of these characters will result in the variable being excluded from the Bayesian network analysis. Metadata should have two columns, one containing the names of your variables and the second containing variable description data. We suggest using gene family or class as a descriptor. All data files must be in CSV format. If you are working in Excel, data tables can be exported to CSV via: File > Save As, then select 'Comma Separated Values (.csv)' from the drop-down menu.

**Figure S2.** Bayesian Networks generated from example genomic datasets. **A–H.** These networks were generated as interactive HTML files, which can be found as downloadable figures at <https://figshare.com/articles/dataset/ReGAIN_command_line_software_and_supplemental_figures_/28959431>.

**Table S3.** Comparison of Bayesian network results across different genomic populations of sizes. ∆ = change. CPR: conditional probability; RR: relative risk; ARD: absolute risk difference. n = number of genomes in example population.

**Table S4.** Comparison of ReGAIN Bayesian network results between an unadjusted genomic population (No Correction) and populations adjusted for genomic similarity at 99.9% and 99.5% average nucleotide identity (ANI). ∆ = change. CPR: conditional probability; RR: relative risk; ARD: absolute risk difference.

**Table S5.** Reference genes used in ReGAIN Curate test dataset. Gene type represents either characterized or predicted resistance. NCBI Accession indicates a closely matching reference gene to the corresponding gene query. Under ‘Identified by AMRfinderPlus’, N/A values are used because AMRfinder does not function to identify transposable elements. Yes = gene was identified using ReGAIN Module 1; No = gene was not identified using ReGAIN Module 1. All ‘No’ and ‘N/A’ annotated genes were positively identified using ReGAIN Curate.

| **Reference Gene Query** | **Identified by AMRfinderPlus** | **Gene Type** | **NCBI Accession** |
| --- | --- | --- | --- |
| *aph(6)-Id* | Yes | Aminoglycoside resistance | WP_250188255.1 |
| *aph(3’’)-Ib* | Yes | Aminoglycoside resistance | HDR0739502.1 |
| *sul1* | Yes | Sulfonamide resistance | EKQ5508431.1 |
| *sul2* | Yes | Sulfonamide resistance | KHI23298.1 |
| *eamA* | No | Small molecule efflux | WP_001390335.1 |
| *tet(A)* | Yes | Tetracycline resistance | ADL14073.1 |
| *mph(A)* | Yes | Macrolide resistance | ENS3968478.1 |
| *mrx(A)* | No | Macrolide resistance | WP_000004159.1 |
| *mphR(A)* | No | Macrolide resistance (operon regulation) | AHX99591.1 |
| *dfrA17* | Yes | Trimethoprim resistance | SRK82092.1 |
| *aadA5* | Yes | Aminoglycoside | STJ94251.1 |
| *aac(3)-IId* | Yes | Aminoglycoside | ESD46483.1 |
| *tmrB* | No | Putative tunicamycin resistance | HBN4657367.1 |
| *chrA* | No | Chromate efflux | ESD86461.1 |
| *qacEdelta1* | Yes | Quaternary ammonium efflux | WP_171455381.1 |
| IS6 family | N/A | Transposase | AOZ86963.1 |
| Transposase | N/A | Transposable element | ACQ42061.1 |
| Shufflon protein D | N/A | Transposable element | WP_250192172.1 |
| *Class-I integron integrase* | N/A | Integrase | WP_001545935.1 |
| *xerD* | N/A | Tyrosine-type recombinase/integrase | AFU91737.1 |

**Figure S3.** ReGAIN Curate performance using parallel processing. Using a dataset of 500 *E. coli* genomes and 20 reference gene sequences (10,000 total queries), ReGAIN Curate was tested using 1, 2, 4, and 8 dedicated CPUs. Processing time is inversely proportional to number of dedicated CPUs

**Table S6**. Genes used to assess accuracy of ReGAIN Curate gene calling. Genes in this table were identified in *E. coli* strain U15A (1). Sequences were extracted from strain U15A and used queries across a random cohort of 500 *E. coli* genomes. Query coverage and percent identity are relative to reference gene annotated by NCBI accession.

| **Gene** | **Description** | **Query Coverage (%)** | **Percent Identity** | **NCBI Accession** |
| --- | --- | --- | --- | --- |
| *aac(3)-IId* | aminoglycoside N-acetyltransferase AAC(3)-IId | 100 | 100 | WP_000557454.1 |
| *aadA5* | ANT(3'')-Ia family aminoglycoside nucleotidyltransferase AadA5 | 100 | 100 | WP_000503573.1 |
| *acrF* | multidrug efflux RND transporter permease subunit AcrF | 100 | 99.52 | AAC76298.1 |
| *aph(3'')-Ib* | aminoglycoside O-phosphotransferase APH(3'')-Ib | 100 | 100 | WP_001082319.1 |
| *aph(6)-Id* | aminoglycoside O-phosphotransferase APH(6)-Id | 100 | 100 | WP_000480972.1 |
| *ariR* | biofilm/acid-resistance regulator AriR | 100 | 97.73 | AAC74250.1 |
| *blaEC* | BlaEC family class C beta-lactamase | 100 | 98.94 | AAZ85965.1 |
| *dfrA17* | trimethoprim-resistant dihydrofolate reductase DfrA17 | 100 | 100 | WP_001389366.1 |
| *emrD* | multidrug efflux MFS transporter EmrD | 100 | 98.99 | EFF04178.1 |
| *emrE* | multidrug efflux SMR transporter EmrE | 100 | 98.18 | CAA77936.1 |
| *fdeC* | inverse autotransporter adhesin FdeC | 100 | 96.75 | ABE05822.1 |
| *iha* | bifunctional siderophore receptor/adhesin Iha | 100 | 100 | ADN47767.1 |
| *iss* | increased serum survival lipoprotein Iss | 100 | 100 | ADN71659.1 |
| *iucA* | aerobactin synthase IucA | 100 | 98.61 | EGI25746.1 |
| *iucB* | N(6)-hydroxylysine O-acetyltransferase IucB | 100 | 99.37 | AAN82074.1 |
| *iucC* | NIS family aerobactin synthetase IucC | 100 | 99.14 | AAN82073.1 |
| *iucD* | NADPH-dependent L-lysine N(6)-monooxygenase IucD | 95.51 | 97.65 | AAD44749.1 |
| *iutA* | ferric aerobactin receptor IutA | 99.73 | 89.85 | AAN45165.2 |
| *mdtM* | multidrug efflux MFS transporter MdtM | 100 | 97.07 | AAC77293.1 |
| *mph(A)* | Mph(A) family macrolide 2'-phosphotransferase | 100 | 100 | WP_000219391.1 |
| *papA* | P fimbria major subunit PapA | 51.89 | 87.5 | CAA43562.1 |
| *qacEdelta1* | quaternary ammonium compound efflux SMR transporter QacE delta 1 | 100 | 100 | WP_000679427.1 |
| *sat* | serine protease autotransporter toxin Sat | 100 | 100 | CAE55775.1 |
| *sul1* | sulfonamide-resistant dihydropteroate synthase Sul1 | 100 | 100 | WP_000259031.1 |
| *sul2* | sulfonamide-resistant dihydropteroate synthase Sul2 | 100 | 100 | WP_001043260.1 |
| *tet(A)* | tetracycline efflux MFS transporter Tet(A) | 100 | 100 | WP_000804064.1 |
| *ybtP* | yersiniabactin ABC transporter ATP-binding/permease protein YbtP | 100 | 99.5 | CAA21388.1 |
| *ybtQ* | yersiniabactin ABC transporter ATP-binding/permease protein YbtQ | 100 | 99.5 | AAC69584.1 |

**Table S7**. Accuracy of gene calling using the ReGAIN Curate pipeline compared to AMRfinderPlus using a cohort of 500 randomly selected *E. coli* genomes. Number of hits represent how many times the gene was identified within the genomic population. Accuracy is defined as ‘(number of hits [AMRfinder]/number of hits [ReGAIN Curate]) * 100’. Values below 100% indicate more hits using ReGAIN Curate; values above 100% indicate more hits using AMRfinderPlus. Genes in bold are described as virulence, stress, or heat tolerance-associated genes. Non-bolded genes are associated with antibiotic or biocide resistance.

| **AMRfinder** | **Number of Hits** | **ReGAIN Curate** | **Number of Hits** | **Accuracy %**  **(≥90% identity, ≥75% query coverage)** | **Number of Hits (≥90% identity, ≥50% query coverage)** | **Accuracy %**  **(≥90% identity, ≥50% query coverage)** |
| --- | --- | --- | --- | --- | --- | --- |
| *aac(3)-IId* | 63 | *aac(3)-IId* | 82;  (63 *aac(3)-IId, 19 aac(3)-IIe* | 76.8 | 83 | 75.9 |
| *aadA5* | 73 | *aadA5* | 67 | 109.0 | 71 | 102.8 |
| *acrF* | 482 | *acrF* | 466 | 103.4 | 482 | 100.0 |
| *aph(3’’)-Ib* | 160 | *aph(3’’)-Ib* | 159 | 100.6 | 160 | 100.0 |
| *aph(6)-Id* | 160 | *aph(6)-Id* | 160 | 100.0 | 160 | 100.0 |
| ***ariR*** | 453 | ***ariR*** | 449 | 100.9 | 453 | 100.0 |
| *blaEC* | 481 | *blaEC* | 481 | 100.0 | 481 | 100.0 |
| *dfrA17* | 81 | *dfrA17* | 82 | 98.8 | 85 | 95.3 |
| *emrD* | 217 | *emrD* | 487 | 44.6 | 487 | 44.6 |
| *emrE* | 345 | *emrE* | 335 | 103.0 | 345 | 100.0 |
| ***fdeC*** | 392 | ***fdeC*** | 391 | 100.3 | 399 | 98.2 |
| ***iha*** | 134 | ***iha*** | 131 | 102.3 | 133 | 100.8 |
| ***iss*** | 242 | ***iss*** | 241 | 100.4 | 241 | 100.4 |
| ***iucA*** | 192 | ***iucA*** | 186 | 103.2 | 186 | 103.2 |
| ***iucB*** | 190 | ***iucB*** | 189 | 100.5 | 192 | 99.0 |
| ***iucC*** | 192 | ***iucC*** | 190 | 101.1 | 192 | 100.0 |
| ***iucD*** | 186 | ***iucD*** | 184 | 101.1 | 186 | 100.0 |
| ***iutA*** | 193 | ***iutA*** | 166 | 116.3 | 167 | 115.6 |
| *mdtM* | 385 | *mdtM* | 384 | 100.3 | 385 | 100.0 |
| *mph(A)* | 135 | *mph(A)* | 135 | 100.0 | 135 | 100.0 |
| ***papA*** | 111 | ***papA*** | 108 | 102.8 | 109 | 101.8 |
| *qacEdelta1* | 158 | *qacEdelta1* | 160 | 98.8 | 160 | 98.8 |
| ***sat*** | 100 | ***sat*** | 80 | 125.0 | 89 | 112.4 |
| *sul1* | 154 | *sul1* | 151 | 102.0 | 153 | 100.7 |
| *sul2* | 172 | *sul2* | 169 | 101.8 | 172 | 100.0 |
| *tet(A)* | 198 | *tet(A)* | 190 | 104.2 | 198 | 100.0 |
| ***ybtP*** | 227 | ***ybtP*** | 226 | 100.4 | 227 | 100.0 |
| ***ybtQ*** | 227 | ***ybtQ*** | 226 | 100.4 | 227 | 100.0 |

1. Forde, B.M., Roberts, L.W., Phan, M.D., Peters, K.M., Fleming, B.A., Russell, C.W., Lenherr, S.M., Myers, J.B., Barker, A.P., Fisher, M.A. *et al.* (2019) Population dynamics of an Escherichia coli ST131 lineage during recurrent urinary tract infection. *Nat Commun*, **10**, 3643.
